# Readiness and motivation to change in the treatment of adults with anorexia nervosa: a case series

**DOI:** 10.1186/2050-2974-2-S1-O8

**Published:** 2014-11-24

**Authors:** Olivia Carter, Susan Byrne, Karina Allen, Anthea Fursland

**Affiliations:** 1 University of Western Australia, Perth, Australia; 2 Centre for Clinical Interventions, Perth, Australia

## 

Lack of motivation and readiness to change is one of the key obstacles to effective treatment of eating disorders, particularly disorders of the anorexic phenotype (anorexia nervosa [AN] or atypical AN). Previous research in this area has suggested the need for more frequent assessment of clients' readiness in order to promote therapeutic intervention that is responsive to their stage of change. The current study sought to achieve this through a case series of 9 clients over the age of 16 being treated for AN or atypical AN through the Centre for Clinical Interventions (public clinic) in Perth, Western Australia. Clients' ages ranged from 17 to 54 with a mean of 27.3 (SD=12.37), and all were female. In addition to measures collected as part of standard clinical care, participants completed the Anorexia Nervosa Stage of Change Questionnaire (ANSOCQ) and the Pros and Cons of Anorexia Nervosa (P-CAN) measure as each treatment session. Trends in fluctuating readiness and motivation to change and variation in client perceptions of the positive and negative aspects of the disorder across the course of treatment were examined, and will be presented on average and for each case. Results may help to inform future practice involving frequent feedback to practitioners about the clients' motivation to change.

This abstract was presented in the **Treatment in Community and Inpatient Settings** stream of the 2014 ANZAED Conference.

